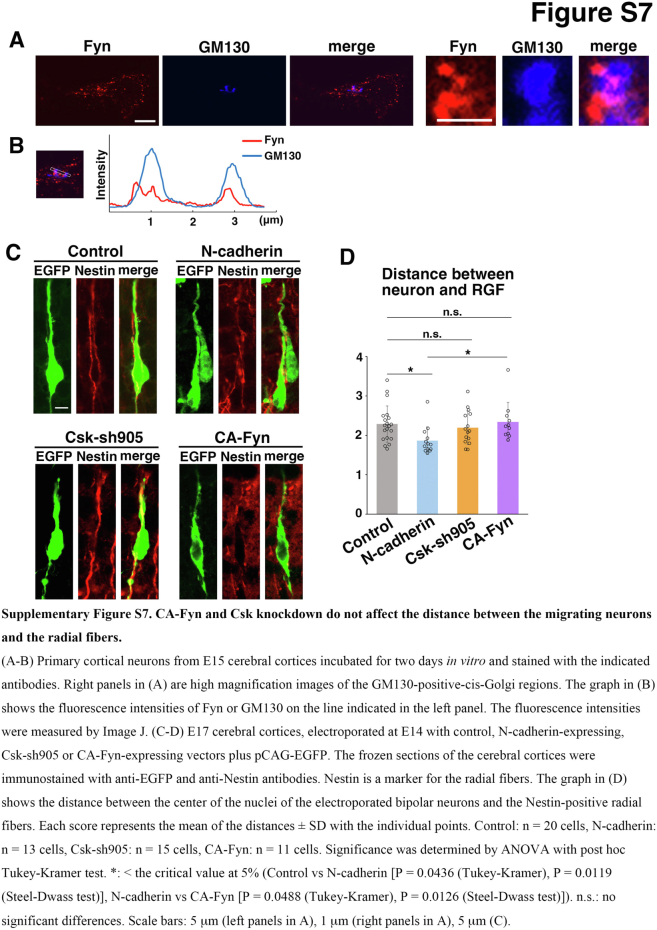# Csk-dependent and -independent control of Src family kinases directs neuronal migration in the developing cerebral cortex

**DOI:** 10.1016/j.jbc.2025.110877

**Published:** 2025-10-30

**Authors:** Yoshiaki V. Nishimura, Shiho Ito, Takeshi Kawauchi

**Affiliations:** 1 Division of Neuroscience, Faculty of Medicine, Tohoku Medical and Pharmaceutical University, Sendai, Miyagi, Japan; 2 Department of Adaptive and Maladaptive Responses in Health and Disease, Graduate School of Medicine, Kyoto University, Medical Innovation Center (3F), Kyoto, Japan; 3 Department of Physiology, Keio University School of Medicine, Tokyo, Japan

**Keywords:** neuronal migration, Csk, Src family kinase, Fyn, cerebral cortex, neural development, N-cadherin, cell adhesion, L1-CAM, immature neurite, neuronal maturation

## Abstract

Fyn, a proto-oncogene product of Src family kinases (SFKs), plays pivotal roles in various pathological and physiological events, including cancer and immunity. C-terminal Src kinase (Csk) is a negative regulator for all SFKs, including Fyn, and acts as a tumor suppressor, but its involvement in normal tissue morphogenesis remains unclear. Here, we show that Csk plays essential roles in cerebral cortical development. *In vivo* knockdown of Csk, as well as constitutive active form of Fyn (CA-Fyn), disturbs neuronal migration and immature neurite formation without affecting neural progenitor proliferation and neuronal differentiation in the developing cerebral cortex. Although overactivation of SFKs is known to downregulate cadherin-mediated cell-to-cell adhesion, both Csk knockdown and CA-Fyn promote attachments between immature neurons. Interestingly, CA-Fyn, but not Csk knockdown, increases N-cadherin cell surface levels. Csk knockdown upregulates SFK activities near the plasma membrane, but not in total cell lysates, whereas CA-Fyn promotes both, implying a Csk-independent role of cytosolic Fyn in N-cadherin plasma membrane localization. In contrast, Csk knockdown and CA-Fyn commonly promote cell surface levels of L1-CAM, an immunoglobulin superfamily cell adhesion molecule that controls cortical neuronal migration. Thus, Csk-mediated local regulation of membrane-bound SFK activities appears essential for cortical neuronal migration through fine-tuning of intercellular attachment strength between immature neurons.

Src family kinases (SFKs) are nonreceptor tyrosine kinases that contain Src homology 3 (SH3), SH2, and kinase domains (1, 2, 3). SFKs consist of at least eight members, including Fyn and Src, and are activated through autophosphorylation in the kinase domain. Activated Fyn and Src can promote cell proliferation and migration through the regulation of cell adhesion, cytoskeletal organization, and signal transduction pathways. Cell adhesion can be classified as cell-to-cell and cell-to-extracellular matrix (ECM) adhesion, where the main cell adhesion molecules are cadherins or integrins, respectively (4, 5). The double knockout of Fyn and Src impairs cadherin-mediated adhesion in keratinocytes, suggesting that the basal activities of Fyn and Src are required for cell-to-cell adhesion (6); however, many studies have reported that overactivation of Fyn or Src disrupts cell-to-cell adhesion through phosphorylation of the cadherin cytoplasmic region and its binding protein, β-catenin, thereby inducing the internalization of cadherins (7, 8, 9, 10, 11, 12, 13, 14). Consistently, the expression levels and/or activities of Fyn or Src are elevated in many cancers, and the activated Fyn or Src promote epithelial–mesenchymal transition and invasion into the basement membrane (3, 9, 15, 16, 17, 18). Overactivation of Fyn and Src appears harmful to multicellular organisms.

C-terminal Src kinase (Csk) is another nonreceptor tyrosine kinase, which phosphorylates the tyrosine residue in the C-terminal region of all SFKs (19, 20, 21). Csk-mediated phosphorylation of SFKs induces intramolecular interaction between the phosphorylated tyrosine residue and the SH2 domain, resulting in suppression of SFK kinase activity. Consistent with the negative effect of Csk on SFKs, Csk is reported to exhibit tumor-suppressing properties (22, 23). In addition to its roles in pathological conditions, Csk is physiologically required for early development, as Csk-deficient mice die at early embryonic stages (24, 25). However, the involvement of Csk in tissue morphogenesis during late developmental stages remains largely unknown.

We and others have reported that Fyn, which is highly expressed in the brain, plays multiple roles in brain development (26, 27, 28). During development of the cerebral cortex in mammals, neural progenitors actively proliferate near the ventricle, generating immature neurons that migrate toward the pial surface (29, 30). The newly generated neurons exhibit multipolar morphologies in the lower part of the intermediate zone (lower-IZ) and subsequently transform into bipolar-shaped neurons (31). Defects in the multipolar-to-bipolar transition have been reported to be associated with several neurological disorders, such as lissencephaly (29). Knockdown of Fyn suppresses the multipolar-to-bipolar transition of immature neurons, and these neurons become stalled in the early phase of neuronal migration (27, 32). The bipolar-shaped neurons, called locomoting neurons, exhibit long-distance migration along neural progenitor–derived radial fibers from the upper region of the IZ toward the top of the cortical plate (CP); this is referred to as a locomotion mode of migration (33, 34). Pharmacological inhibition of SFKs delays the migration speed of the locomoting neurons, suggesting that SFK activities are required for the long-distance neuronal migration (27). At the final phase of the migration, neurons detach from the radial fibers and exhibit a somal movement with retraction of the leading process (terminal translocation mode of migration) (35). Reelin, a secreted large molecule, has been shown to be required for the formation of the mammalian-specific six-layered structure of the cerebral cortex. Binding of reelin to its receptors induces Fyn- and Src-mediated phosphorylation of Dab1, a cytoplasmic adaptor protein, to activate downstream signaling pathways (36, 37, 38). The reelin–Dab1 signals promote the terminal translocation through activation of α5β1–integrins, suggesting that reelin-mediated activation of SFKs is important for cell-to-ECM adhesion in the final phase of neuronal migration (39). In contrast, the upstream regulatory molecules of Fyn and Src in the early or middle phases of neuronal migration remain unclear.

Fyn and Src are proto-oncogene products, and thus, their activities should be tightly regulated during normal tissue morphogenesis to avoid their overactivation. We therefore focused on the role of Csk in cerebral cortical development. In this study, we revealed that Csk plays essential roles in the early phase of neuronal migration. Interestingly, distinct from many non-neuronal cultured cells (8, 9, 11, 12), *in vivo* overactivation of Fyn promotes cell-to-cell adhesion and increases cell surface levels of N-cadherin and L1-CAM, an immunoglobulin superfamily cell adhesion molecule. In contrast, knockdown of Csk, which increases SFK activities only near the plasma membrane, promotes cell surface levels of L1-CAM, but not N-cadherin, suggesting Csk-dependent and -independent regulation of cell adhesion molecules in immature neurons, which appears essential for neuronal migration in the developing cerebral cortex.

## Results

### Overactivation of Fyn perturbs neuronal migration and enhances neuronal attachment

**Figure 1:**
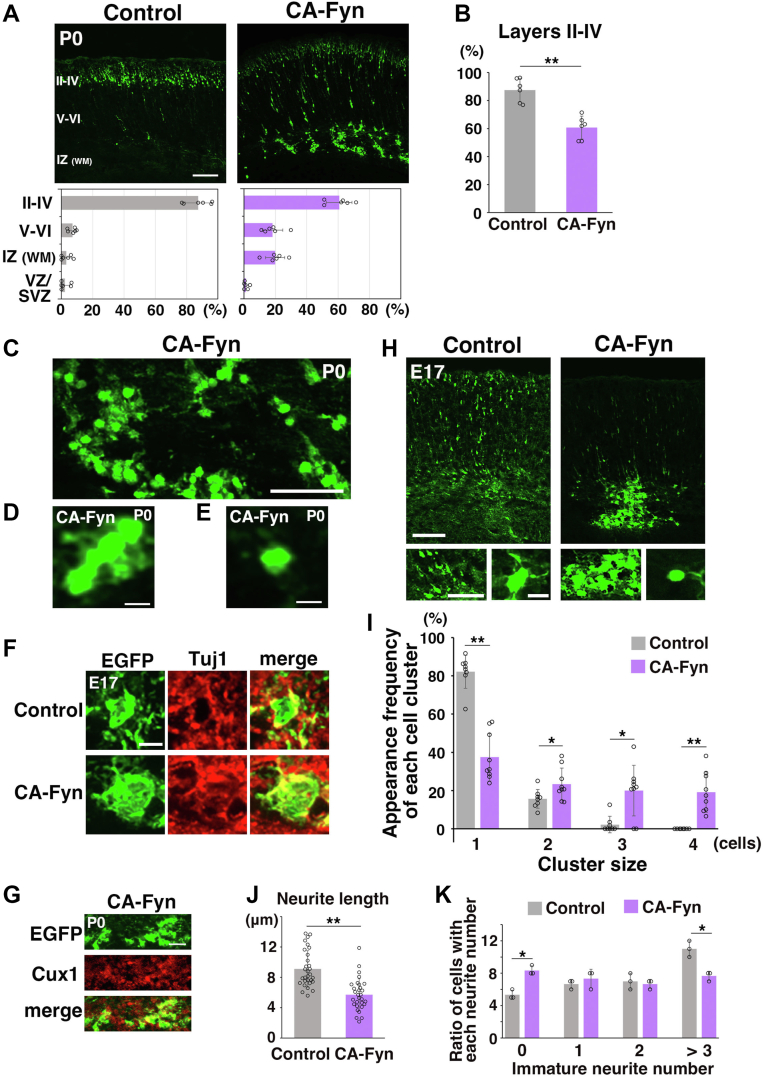
**Overactivation of Fyn perturbs neuronal migration and enhances immature neuronal attachments.***A* and *B*, cerebral cortices at P0, electroporated at E14 with control or CA-Fyn-expressing vectors plus pCAG-EGFP. The *lower graphs* in (*A*) calculate cell migration by quantifying EGFP-positive cell numbers in distinct regions of the cerebral cortices. Each score represents the mean of the ratios ± SD with the individual points. The *graph* in (*B*) shows the ratio of the number of the electroporated cells in the *upper layers* (layers II–IV). Each score represents the mean of the ratios ± SD with the individual points. Control: n = 6 brains, CA-Fyn: n = 6 brains. Significance compared with control was determined by Student’s *t* test (II–IV, V–VI, and IZ) or Mann–Whitney *U* test (VZ/SVZ). CA-Fyn (layer II–IV): *p* = 0.000234 (Student’s *t* test), CA-Fyn (layer V–VI): *p* = 0.00304 (Student’s *t* test), CA-Fyn (IZ [WM]): *p* = 0.000170 [Student’s *t* test], CA-Fyn (VZ/SVZ): *p* = 0.868 [Mann–Whitney *U* test], *p* = 0.502 [Student’s *t* test]. ∗∗*p* < 0.01. *C*–*E*, high-magnification images at the IZ of P0 cerebral cortices that were electroporated at E14 with CA-Fyn-expressing vectors plus pCAG-EGFP. CA-Fyn-electroporated cells formed clusters (*D*) and exhibited round morphologies (*E*). *F*, E17 cerebral cortices, electroporated at E14 with control or CA-Fyn-expressing vectors plus pCAG-EGFP. The frozen sections of the cerebral cortices were immunostained with anti-EGFP and anti-Tuj1 (βIII-tubulin) antibodies. The entire images of the cortical layers are shown in Fig. S1*A*. *G*, P0 cerebral cortices, electroporated at E14 with control or CA-Fyn-expressing vectors plus pCAG-EGFP. The frozen sections of the cerebral cortices were immunostained with anti-EGFP and anti-Cux1 antibodies. The entire images with DAPI staining of the cortical layers are shown in Fig. S1*B*. *H*–*K*, E17 cerebral cortices, electroporated at E14 with control or CA-Fyn-expressing vectors plus pCAG-EGFP. The *lower panels* in (*H*) are high magnification images of the IZ. Increased attachment between immature neurons (*lower left panels*) and suppression of immature neurite formation (*lower right panels*) were observed. The *graph* in (*I*) shows the ratio of the number of each small cell cluster that contains one, two, three, or four cells. Each score represents the mean of the ratios ± SD with the individual points. Control: n = 8 brains, CA-Fyn: n = 9 brains. Significance compared with control was determined by Student’s *t* test (cluster size = 2) or Mann–Whitney *U* test (cluster size = 1, 3, and 4). CA-Fyn (cluster size = 1): *p* = 0.000000425 (Student’s *t* test), *p* = 0.0000823 (Mann–Whitney *U* test). CA-Fyn (cluster size = 2): *p* = 0.0386 (Student’s *t* test), *p* = 0.0360 (Mann–Whitney *U* test). CA-Fyn (cluster size = 3): *p* = 0.00247 (Student’s *t* test), *p* = 0.0127 (Mann–Whitney *U* test). CA-Fyn (cluster size = 4): *p* = 0.000128 (Student’s *t* test), *p* = 0.000255 (Mann–Whitney *U* test). ∗*p* < 0.05, ∗∗*p* < 0.01. The *graph* in (*J*) shows the length of the immature neurites of the electroporated cells in the IZ. The lengths were measured by Photoshop CS6 Extended (Adobe). Each score represents the mean of the lengths (μm) ± SD with the individual points. Control: n = 30 cells, CA-Fyn: n = 30 cells. Significance compared with control was determined by Mann–Whitney *U* test. CA-Fyn: *p* = 0.000000604. ∗∗*p* < 0.01. The *graph* in (*K*) shows the ratio of the number of cells with 0, 1, 2, or >3 immature neurites. Each score represents the mean of the ratios ± SD with the individual points. Control: n = 3 brains (90 cells), CA-Fyn: n = 3 brains (90 cells). Significance compared with control was determined by Mann–Whitney *U* test. CA-Fyn (cells without immature neurite): *p* = 0.0431 (Mann–Whitney *U* test). CA-Fyn (cells with one immature neurite): *p* = 0.361 (Mann–Whitney *U* test). CA-Fyn (cells with two immature neurites): *p* = 0.637 (Mann–Whitney *U* test). CA-Fyn (cells with more than three immature neurites): *p* = 0.0463 (Mann–Whitney *U* test). ∗*p* < 0.05. The scale bars represent 100 μm (*A*, *upper panels* in *H*), 50 μm (*C*, *lower left panels* in *H*), 12.5 μm (*D*, *E*), 5 μm (*F*), 25 μm (*G*), 10 μm (*lower right panels* in *H*). II–IV, layers II–IV of the cortical plate; V–VI, layers V–VI of the cortical plate; CA-Fyn, constitutive active form of Fyn; DAPI, 4′,6-diamidino-2-phenylindole dihydrochloride; E14, embryonic day 14; EGFP, enhanced GFP; IZ, intermediate zone; SVZ, subventricular zone; VZ, ventricular zone; WM, white matter.

We first analyzed the effect of overactivation of SFKs on cerebral cortical development *in vivo*. To test this, we transferred plasmids encoding a constitutively active form of Fyn, a major SFK isoform in the developing cerebral cortex (constitutive active form of Fyn [CA-Fyn]), and enhanced GFP (EGFP) into mouse cerebral cortices at embryonic day 14 (E14) using an *in vivo* electroporation method (40). As reported previously (40), most EGFP-positive electroporated cells reached the superficial layer of the CP in control brains at postnatal day 0 (P0), 5 days after the electroporation (Fig. 1*A*). In contrast, CA-Fyn-expressing cells exhibited defects in the migration toward the pial surface, and these cells remained stalled mainly at the IZ at P0 (Fig. 1*A*). The ratio of cells in the upper layers (layer II–IV) of the CP was significantly decreased in the CA-Fyn-electroporated cortices (Fig. 1*B*). To our surprise, CA-Fyn-expressing cells formed large aggregates or were observed linked together like beads, suggesting an increase in cell adhesion (Fig. 1, *C* and *D*). Moreover, individual CA-Fyn-expressing cells displayed round morphologies lacking immature neurites (Fig. 1*E*).

**Supporting Figure S1:**
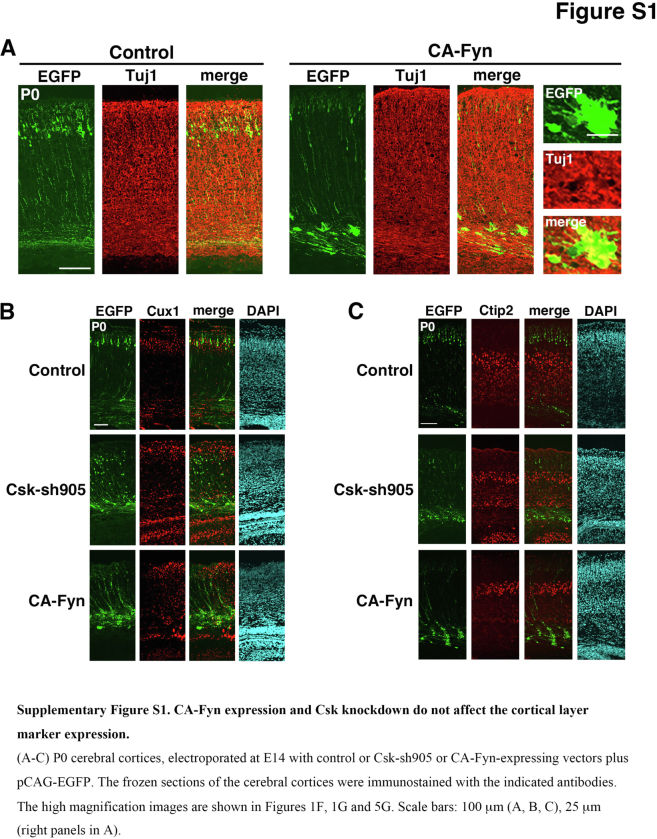


Because the EGFP-positive cells in control cerebral cortices have completed their migration and were rarely observed in the IZ at P0, we chose an earlier timepoint for examination, E17 (3 days postelectroporation), when most of the electroporated cells are located in the IZ in the control cerebral cortices (40). At E17, both control and CA-Fyn-expressing cells were observed in the IZ and were positive for Tuj1, a marker for postmitotic neurons, and these neurons subsequently expressed Cux1, but not Ctip2, markers for the upper or deep layer neurons, respectively, suggesting that CA-Fyn does not affect the neuronal differentiation (Figs. 1, *F*, *G* and S1, *A*–*C*). However, in contrast to the control cells in the IZ, CA-Fyn-expressing cells frequently formed clusters (Fig. 1*H*). The ratio of cells with no attachment to neighboring cells was decreased in the CA-Fyn-electroporated cortices (“cluster size = 1 cell” in Fig. 1*I*). In contrast, many CA-Fyn-electroporated cells formed small clusters in the IZ (“cluster size = 2, 3, or 4 cells” in Fig. 1*I*). In addition, CA-Fyn-expressing cells exhibited round morphologies, whereas control cells in the IZ extended many immature neurites (*lower right panels* in Fig. 1*H*). The average length of the immature neurites and the ratio of cells with more than three immature neurites were significantly decreased in the CA-Fyn-expressing neurons (Fig. 1, *J* and *K*). These results indicate that CA-Fyn enhances the attachment between immature neurons *in vivo*, although it has been reported that overactivation of SFKs reduces cell-to-cell adhesion in many cultured cells (8, 11, 13).

### Csk is expressed in the IZ and CP in the developing mouse cerebral cortex

Increased neuronal attachments by overexpression of CA-Fyn suggest that Fyn has a positive effect on cell-to-cell adhesion of neurons *in vivo*. Therefore, we next examined physiological relationships between increased SFK activities and cell adhesion properties. To test this, we focused on Csk, an endogenous negative regulator for all SFKs, including Fyn, because suppression of Csk is expected to activate SFKs within the range of the endogenous regulation by the upstream regulator.

**Figure 2:**
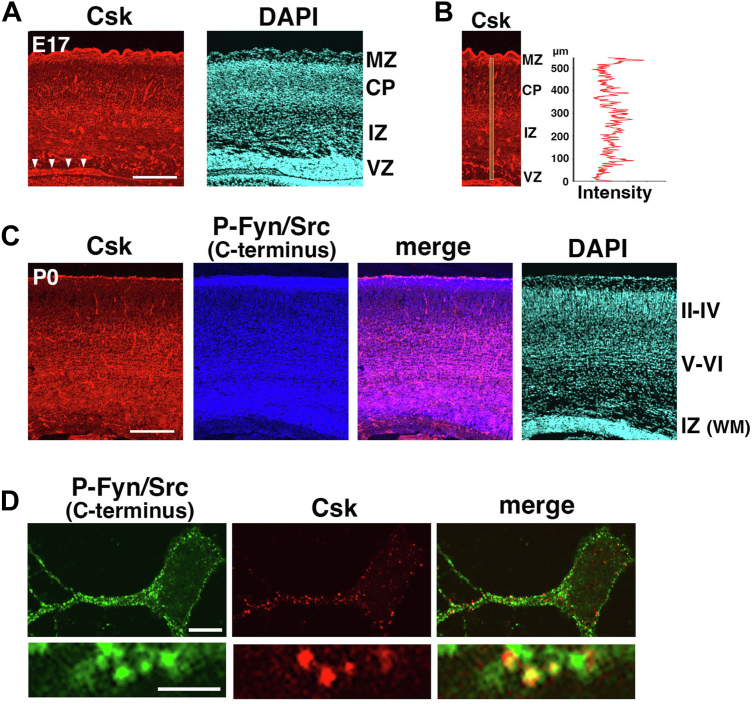
**Expression patterns of Csk in the developing cerebral cortex.***A* and *B*, cryosections of E17 cerebral cortex immunostained with anti-Csk antibody *(red*) and DAPI (*light blue*). The *graph* in (*B*) shows the fluorescence intensities of Csk on the line indicated in the *left panel*. The fluorescence intensities were measured by FV-10i software (FLUOVIEW; Olympus). Note that the fluorescence intensities in 0 to 70 μm (corresponding to the VZ) were lower than the other regions. *Arrowheads* indicate the choroid plexus distinct from the cerebral cortex. *C*, cryosections of P0 cerebral cortex immunostained with anti-Csk (*red*) and anti-phosphorylated Fyn/Src at the C terminus (Y530P-Src) (*blue*) antibodies and DAPI (*light blue*). *D*, primary cortical neurons from E15 cerebral cortices were incubated for 2 days *in vitro* and immunostained with anti-phosphorylated Fyn/Src at the C terminus (Y530P-Src) (*green*) and anti-Csk (*red*) antibodies. The scale bars represent 200 μm (*A*, *C*), 5 μm (*upper panels* in *D*), and 1 μm (*lower panels* in *D*). II–IV, layers II–IV of the cortical plate; V–VI, layers V–VI of the cortical plate; E17, embryonic day 17; CP, cortical plate; Csk, C-terminal Src kinase; DAPI, 4′,6-diamidino-2-phenylindole dihydrochloride; IZ, intermediate zone; MZ, marginal zone; VZ, ventricular zone; WM, white matter.

We first analyzed the expression pattern of Csk in the developing mouse cerebral cortex. Immunohistochemistry revealed that Csk was expressed throughout the cerebral cortex at E17 (Fig. 2*A*). Stronger expression was detected in the IZ and the deep layers of the CP, where immature neurons actively migrate, whereas lower expression was observed in the ventricular zone (VZ), where neural progenitors are located (Fig. 2*B*). At P0, relatively strong expression was observed in the white matter (corresponding to the IZ in embryonic brains) and deep layers (layers V and VI) of the cerebral cortex (Fig. 2*C*). Csk is known to phosphorylate SFKs at the C-terminal region to suppress their tyrosine kinase activities. Antibody detection for the phosphorylated Fyn and Src at the C-terminal regions revealed similar patterns to that of Csk (Fig. 2*C*). To examine this at the subcellular level, we performed immunocytochemistry of primary cultures of neurons from E15 embryonic cerebral cortices. At 2 days *in vitro* (DIV), we observed that the Fyn and Src phosphorylated at the C terminus were colocalized with Csk in primary cortical neurons (Fig. 2*D*). Thus, Csk is expressed mainly in the IZ and deep layers of the CP in the developing cerebral cortex, where immature neurons actively migrate, and colocalized with Csk-mediated phosphorylation of Fyn and Src in immature neurons.

**Supporting Figure S2:**
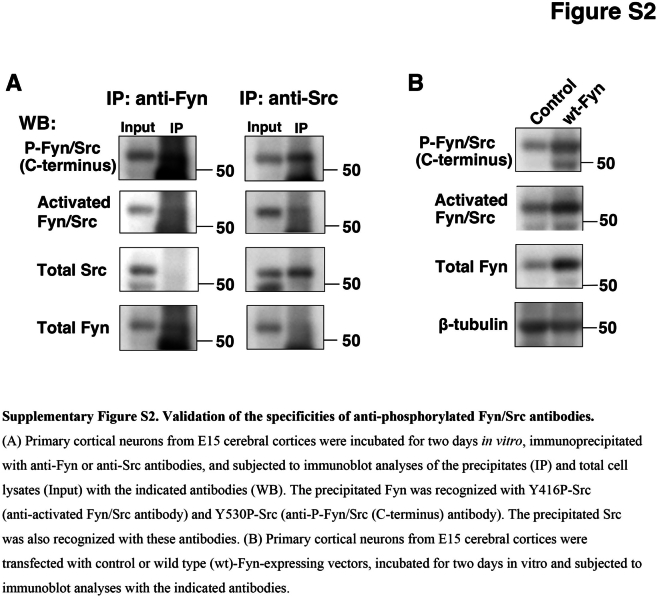


To confirm the specificities of our antibodies, we performed immunoprecipitation experiments using specific antibodies for Fyn or Src. The antibody for Fyn specifically precipitated Fyn, but not Src, and anti-Src antibody precipitated only Src, but not Fyn. Importantly, these precipitants containing either Fyn or Src alone were recognized with both anti-phosphorylated Src at the C terminus and antiactivated Src antibodies (anti-Y530P-Src [CST; #2105] and anti-Y416P-Src [CST; #6943], respectively), indicating that these two antibodies can recognize not only Src but also Fyn (Fig. S2*A*). Consistent with this, the band intensities of immunoblot analyses with these antibodies were increased when wt-Fyn was overexpressed (Fig. S2*B*). Therefore, we refer to these antibodies as antibodies against activated Fyn/Src or P-Fyn/Src (C terminus) in this article.

### Csk suppresses SFK activities only near the plasma membrane

**Figure 3:**
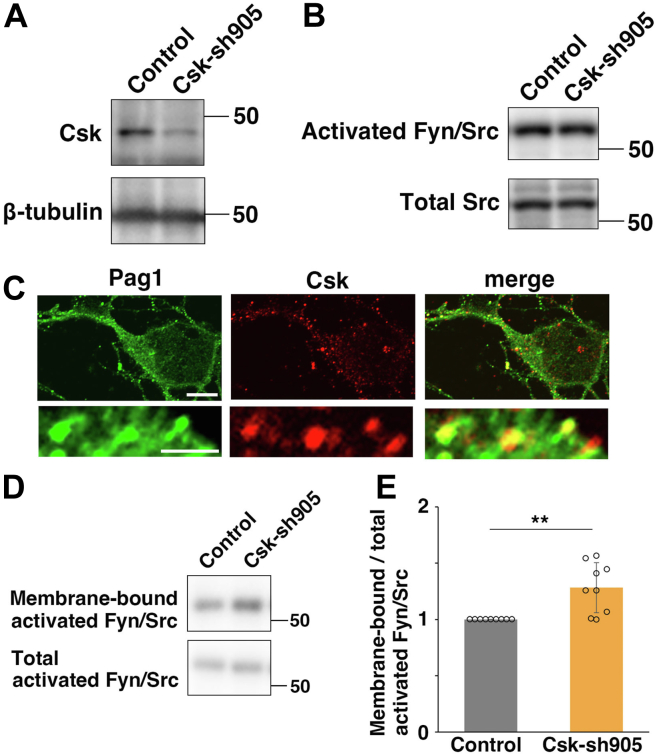
**Csk knockdown increases SFK activities only near the plasma membrane.***A* and *B*, primary cortical neurons from E15 cerebral cortices were transfected with control or Csk-sh905 vectors, incubated for 5 days *in vitro*, and cell lysates were processed for immunoblot analyses with the indicated antibodies. *C*, primary cortical neurons from E15 cerebral cortices were incubated for 2 days *in vitro* and immunostained with anti-Pag1 (*green*) and anti-Csk (*red*) antibodies. *D* and *E*, primary cortical neurons from E15 cerebral cortices were transfected with control or Csk-sh905 vectors, incubated for 2 days *in vitro*, and subjected to cell surface biotinylation assays (see the Experimental procedures section). Immunoblot analyses of the precipitates (biotinylated cell surface proteins and their associated membrane-bound proteins) and total cell lysates with antiactivated Fyn/Src (Y416P-Src) antibody were performed. The graph in (*E*) shows the mean ratios of immunoblot band intensities of membrane-bound activated Fyn/Src to that of total activated Fyn/Src ± SD with the individual points. Significance was determined by a one-sample *t* test. *p* = 0.00509 (n = 9 biological replicates). ∗∗*p* < 0.01. The scale bars represent 5 μm (*upper panels* in *C*), 1 μm (*lower panels* in *C*). Csk, C-terminal Src kinase; DAPI, 4′,6-diamidino-2-phenylindole dihydrochloride; E15, embryonic day 15; EGFP, enhanced GFP; IZ, intermediate zone; SFK, Src family kinase.

**Supporting Figure S3:**
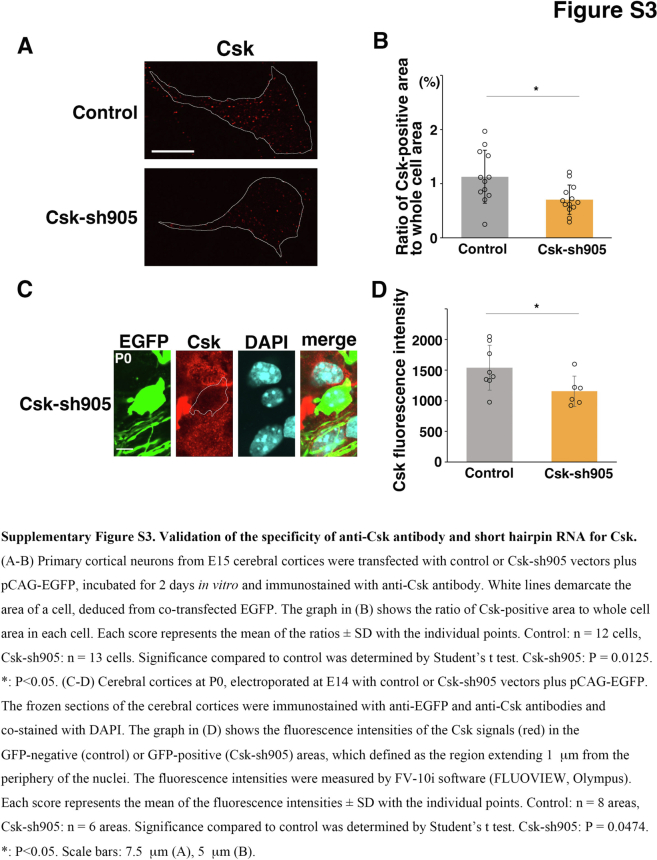


**Supporting Figure S4:**
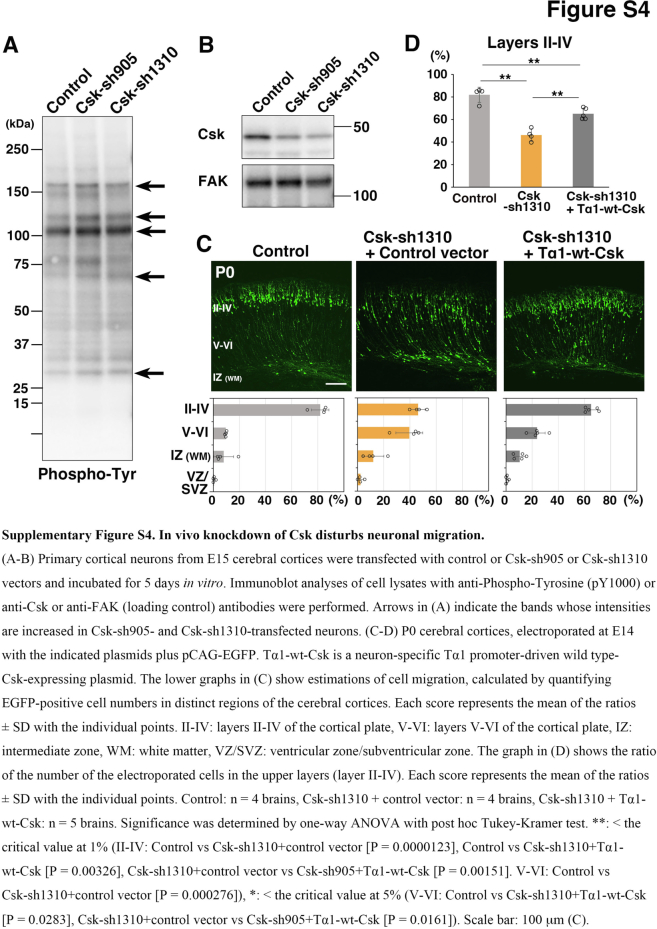


To examine Csk-mediated endogenous regulation of SFK activities in a physiological context, we performed knockdown experiments. We constructed shRNAs targeting a coding sequence of Csk (Csk-sh905). When transfected into primary cortical neurons or cerebral cortices at E14, Csk-sh905 efficiently reduced endogenous Csk protein levels (Figs. 3*A* and S3, *A*–*D*). Consistent with the fact that Csk is a negative regulator for SFKs, our immunoblot analyses with antiphosphotyrosine antibody revealed that Csk-sh905 increased the intensities of several bands representing tyrosine residue–phosphorylated proteins, suggesting upregulation of tyrosine kinase activities in the Csk-sh905–transfected neurons (Fig. S4*A*).

We next examined the effect of Csk knockdown on Fyn and Src activation. Active forms of Fyn and Src undergo autophosphorylation in the kinase domain. Surprisingly, we found that Csk-sh905 had little effect on the activated Fyn/Src in total cell lysates (Fig. 3*B*). Previous studies indicate that Csk is recruited to the plasma membrane by binding to Pag1 (also known as Csk-binding protein) (41, 42). Consistent with this notion, Csk was found to be colocalized with Pag1 in primary cortical neurons (Fig. 3*C*), implying that Csk locally controls Fyn and Src activities. To detect the Fyn and Src activities near the plasma membrane, we treated primary cortical neurons with a membrane-impermeable biotinylation reagent, sulfo-*N*-hydroxy-succinimide-biotin (sulfo-NHS–biotin), to label the cell surface proteins with biotin. The biotinylated cell surface or transmembrane proteins (cell surface proteins) and their associated cytoplasmic proteins that are localized near the plasma membrane (membrane-bound proteins) were precipitated with streptavidin-conjugated beads and subjected to immunoblot analyses with an antibody against activated Fyn/Src. The activated Fyn/Src were detected in the precipitated protein lysates, as well as total cell lysates, suggesting that a portion of SFKs bound to cell surface proteins and localized near the plasma membrane (referred to as “membrane-bound Fyn/Src”). The ratio of the membrane-bound activated Fyn/Src to total activated Fyn/Src was significantly increased in the Csk-sh905–transfected primary cortical neurons (Fig. 3, *D* and *E*). These data indicate that Fyn/Src activities are locally increased near the plasma membrane in Csk-knockdown neurons.

### Csk regulates neuronal migration and morphological changes *in vivo*

**Figure 4:**
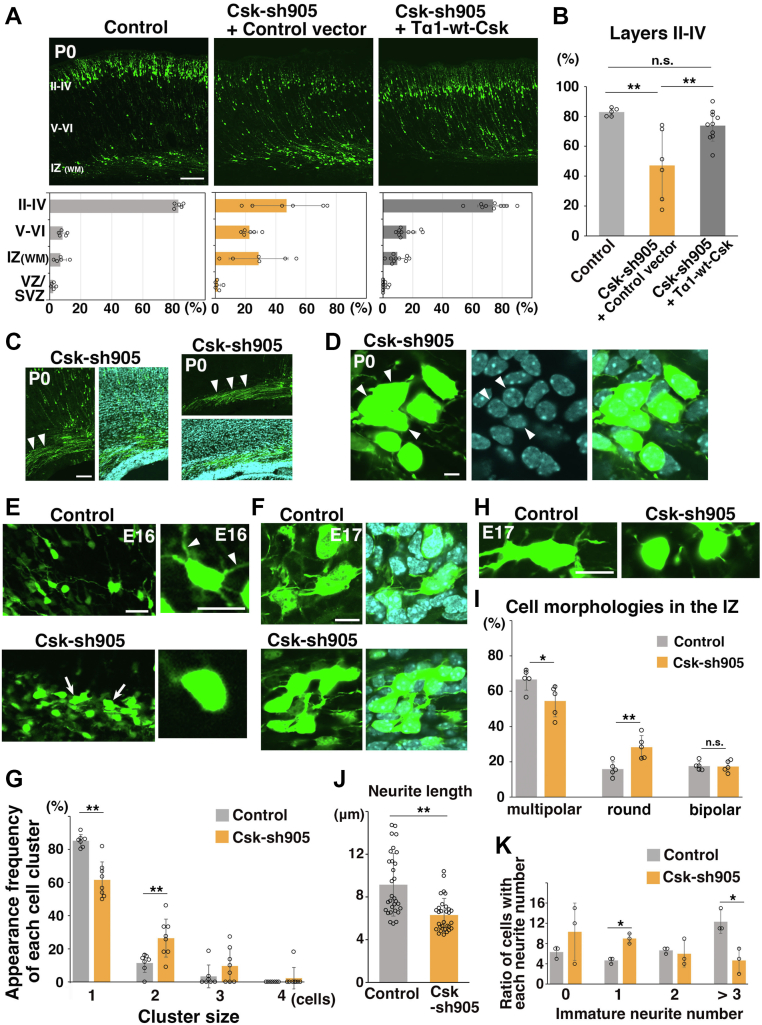
***In vivo* knockdown of Csk disturbs neuronal migration and morphological changes and increases immature neuronal attachments.***A* and *B*, P0 cerebral cortices, electroporated at E14 with the indicated plasmids plus pCAG-EGFP. Tα1-wt-Csk is a neuron-specific Tα1 promoter–driven wt-Csk-expressing plasmid. The *lower graphs* in (*A*) show estimations of cell migration, calculated by quantifying EGFP-positive cell numbers in distinct regions of the cerebral cortices. Each score represents the mean of the ratios ± SD with the individual points. The *graph* in (*B*) shows the ratio of the number of the electroporated cells in the u*pper layers* (layers II–IV). Each score represents the mean of the ratios ± SD with the individual points. Control: n = 5 brains, Csk-sh905 + control vector: n = 6 brains, Csk-sh905 + Tα1-wt-Csk: n = 10 brains. Significance was determined by one-way ANOVA with the post hoc Tukey–Kramer test. ∗∗less than the critical value at 1% (II–IV, control *versus* Csk-sh905 + control vector [*p* = 0.00441, Tukey–Kramer, *p* = 0.0284 [Steel–Dwass test], Csk-sh905 + control vector *versus* Csk-sh905 + Tα1-wt-Csk [*p* = 0.00747, Tukey–Kramer, *p* = 0.0449 [Steel–Dwass test]). V–VI, control *versus* Csk-sh905 + control vector (*p* = 0.00373 [Tukey–Kramer], *p* = 0.0284 [Steel–Dwass test]), ∗less than the critical value at 5% (IZ: control *versus* Csk-sh905 + control vector *p* = 0.0172 [Tukey–Kramer], *p* = 0.133 [Steel–Dwass test]), Csk-sh905 + control vector *versus* Csk-sh905 + Tα1-wt-Csk *p* = 0.0122 [Tukey–Kramer], *p* = 0.124 [Steel–Dwass test]). *C* and *D*, cryosections of P0 cerebral cortices, electroporated at E14 with Csk-sh905 plus pCAG-EGFP, were immunostained with anti-EGFP (*green*) and DAPI (*light blue*). *Arrowheads* in (*C*) indicate axon bundles in two representative Csk-sh905–electroporated cerebral cortices, and *arrowheads* in (*D*) indicate small cell clusters of the Csk-sh905–electroporated cells. *Right panels* in (*C*) are the high magnification images of the medial side of the same cerebral cortex as Fig. S5*A*. *E*, E16 cerebral cortices were electroporated at E14 with control or Csk-sh905 vectors plus pCAG-EGFP. *Right panels* are high magnification images of multipolar or round cells in the IZ of control- or Csk-sh905–electroporated cerebral cortex, respectively. *Arrowheads* in the *right upper panel* indicate immature neurites. Note that less immature neurites were observed in the Csk-sh905–electroporated cells (*right lower panel*). *Arrows* in the *left lower panel* indicate small clusters of Csk-sh905–electroporated cells. *F*–*K*, cryosections of E17 cerebral cortices, electroporated at E14 with control or Csk-sh905 vectors plus pCAG-EGFP, were immunostained with anti-EGFP (*green*) and DAPI (*light blue*). The *graph* in (*G*) shows the ratio of the number of each small cell cluster that contains one, two, three, or four cells. Each score represents the mean of the ratios ± SD with the individual points. Control: n = 7 brains, Csk-sh905: n = 8 brains. Significance compared with control was determined by Student’s *t* test (cluster size = 2), Welch’s *t* test (cluster size = 1), or Mann–Whitney *U* test (cluster size = 3, 4). Csk-sh905 (cluster size = 1): *p* = 0.000268 (Welch’s *t* test), *p* = 0.00124 (Mann–Whitney *U* test). Csk-sh905 (cluster size = 2): *p* = 0.00782 (Student’s *t* test), *p* = 0.00780 (Welch’s *t* test), *p* = 0.00767 (Mann–Whitney *U* test). Csk-sh905 (cluster size = 3): *p* = 0.190 (Welch’s *t* test), *p* = 0.223 (Mann–Whitney *U* test). Csk-sh905 (cluster size = 4): *p* = 0.351 (Welch’s *t* test), *p* = 0.350 (Mann–Whitney *U* test). ∗∗*p* < 0.01. The *graph* in (*I*) shows the ratio of cells with the indicated morphologies in the IZ. Each score represents the mean of the ratios ± SD with the individual points. Control: n = 5 brains, Csk-sh905: n = 5 brains. Significance compared with control was determined by Student’s *t* test. Csk-sh905 (*multipolar*): *p* = 0.0350; Csk-sh905 (*round*): *p* = 0.00736; Csk-sh905 (*bipolar*): *p* = 0.905. ns, no significant differences, ∗*p* < 0.05, ∗∗*p* < 0.01. The *graph* in (*J*) shows the length of the immature neurites of the electroporated cells in the IZ. The lengths were measured by Photoshop CS6 Extended (Adobe). Each score represents the mean of the lengths (μm) ± SD with the individual points. Control: n = 30 cells, Csk-sh905: n = 30 cells. Significance compared with control was determined by Mann–Whitney *U* test. Csk-sh905: *p* = 0.0000251. ∗∗*p* < 0.01. The *graph* in (*K*) shows the ratio of the number of cells with 0, 1, 2, or >3 immature neurites. Each score represents the mean of the ratios ± SD with the individual points. Control, n = 3 brains (90 cells); Csk-sh905, n = 3 brains (90 cells). Significance compared with control was determined by Mann–Whitney *U* test. Csk-sh905 (cells without immature neurites): *p* = 0.507; Csk-sh905 (cells with one immature neurite): *p* = 0.0463; Csk-sh905 (cells with two immature neurites): *p* = 0.507; Csk-sh905 (cells with more than three immature neurites): *p* = 0.0463. ∗*p* < 0.05.The scale bars represent 100 μm (*A*, *C*), 5 μm (*D*), 20 μm (*left panels* in *E*), 10 μm (*right panels* in *E*), 10 μm (*F*, *H*). II–IV, layers II–IV of the cortical plate; V–VI, layers V–VI of the cortical plate; Csk, C-terminal Src kinase; DAPI, 4′,6-diamidino-2-phenylindole dihydrochloride; EGFP, enhanced GFP; SVZ subventricular zone; VZ, ventricular zone; WM, white matter.

Given that Csk-sh905 efficiently reduced endogenous Csk protein levels and increased Fyn/Src activities near the plasma membrane, we next performed *in vivo* knockdown experiments. We electroporated Csk-sh905 into E14 embryonic cerebral cortices. At P0, 5 days after the electroporation, Csk-sh905–electroporated cells were located in both the CP and the IZ, whereas control cells were mainly observed at the upper layers of the CP (Fig. 4*A*). The ratio of cells in the upper layers (layers II–IV) was significantly decreased in the Csk-sh905–electroporated brains (Fig. 4*B*). The migration defects were rescued by coelectroporation with a wt-Csk driven by a neuron-specific Tα1 promoter, suggesting that neuronal Csk plays an important role in the pia-directed migration (Fig. 4, *A* and *B*). We used another knockdown vector, Csk-sh1310, to confirm these results (Fig. S4*B*). As expected, the migration defects of Csk-sh1310–electroporated cells were observed (Fig. S4*C*) and could be partially rescued by coelectroporation with Tα1-wt-Csk (Fig. S4, *C* and *D*).

**Supporting Figure S5:**
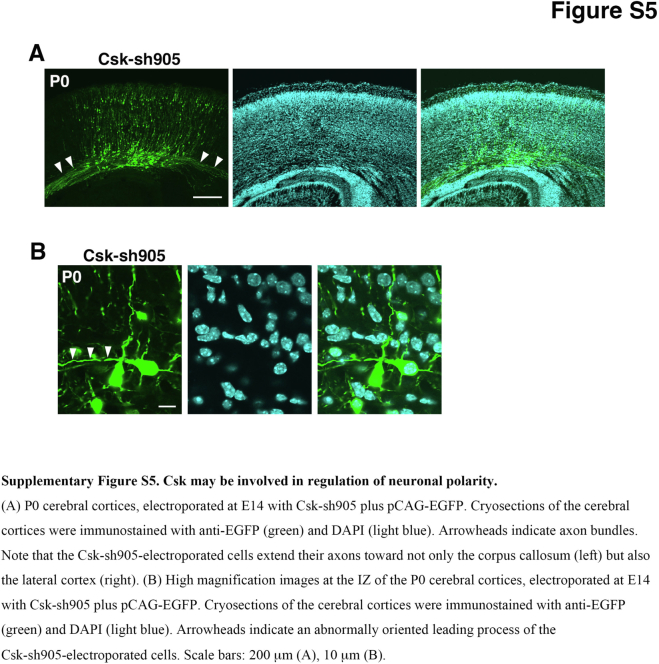


Despite the delayed migration, Csk-sh905–electroporated neurons were able to extend the axons toward the contralateral hemisphere (Fig. 4*C*). This is consistent with previous studies showing EGFP-positive cells that were labeled at E14 become upper layer neurons, which extend their axons toward the contralateral hemisphere *via* the corpus callosum (43, 44). Notably, the Csk-sh905–electroporated cells expressed Cux1, but not Ctip2, suggesting that these cells acquire the properties of upper layer neurons (Figs. S1, *B*, *C* and 5*G*). However, in rare cases, a few Csk-knockdown cells were observed with axons elongating to the opposite (ipsilateral) side or a leading process with a tangential direction, implying that Csk knockdown may affect neuronal polarity (Fig. S5, *A* and *B*).

**Figure 5:**
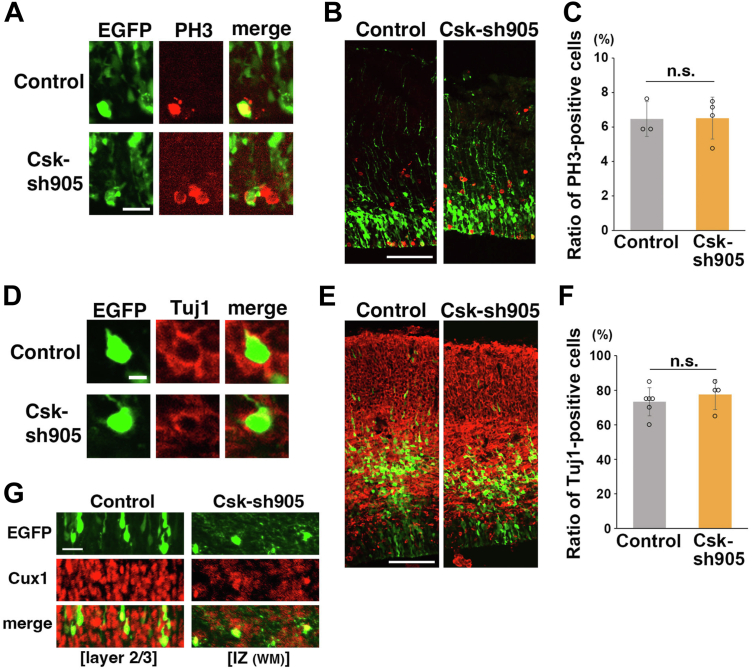
***In vivo* knockdown of Csk does not affect neural progenitor proliferation and neuronal differentiation.***A*–*C*, E15 cerebral cortices, electroporated at E14 with control and Csk-sh905 vectors plus pCAG-EGFP. Brains were fixed 20 h after the electroporation, and the frozen sections were immunostained with anti-EGFP and anti–phospho-Histone H3 (PH3) antibodies. The full cortical layers are shown in (*B*). The *graph* in (*C*) shows the ratio of PH3-positive cells in the electroporated cells in the VZ. Each score represents the mean of ratios ± SD with the individual points. Control: n = 3 brains, Csk-sh905: n = 4 brains. Significance compared with control was determined by Student’s *t* test. Csk-sh905: *p* = 0.960. ns, no significant differences. *D*–*F*, E16 cerebral cortices, electroporated at E14 with control and Csk-sh905 vectors plus pCAG-EGFP. Brains were fixed 50 to 55 h after the electroporation, and the frozen sections were immunostained with anti-EGFP and anti-Tuj1 (βIII-tubulin) antibodies. The entire images of the cortical layers are shown in (*E*). The *graph* in (*F*) shows the ratio of Tuj1-positive cells in the electroporated cells in the IZ. Each score represents the mean of ratios ± SD with the individual points. Control: n = 6 brains, Csk-sh905: n = 4 brains. Significance compared with control was determined by Student’s *t* test. Csk-sh905: *p* = 0.462. ns, no significant differences. *G*, P0 cerebral cortices, electroporated at E14 with control or Csk-sh905 vectors plus pCAG-EGFP. The frozen sections of the cerebral cortices were immunostained with anti-EGFP and anti-Cux1 antibodies. The entire images with DAPI staining of the cortical layers are shown in Fig. S1*B*. The scale bars represent 20 μm (*A*), 100 μm (*B*, *E*), 5 μm (*D*), 25 μm (*G*). Csk, C-terminal Src kinase; DAPI, 4′,6-diamidino-2-phenylindole dihydrochloride; E14, 15, 16, embryonic day 14/15/16; EGFP, enhanced GFP; IZ, intermediate zone; VZ, ventricular zone.

Although Csk-knockdown cells were not usually observed to form large aggregates, small clusters of the electroporated cells were often observed in the Csk-sh905–electroporated brains at P0 (Fig. 4*D*). In addition, Csk-knockdown cells exhibit relatively round morphologies with few or no immature neurites. These phenotypes were similar to CA-Fyn-expressing cells in the IZ (Figure 1, Figure 4, *C*–*E* and 4*D*). To compare the phenotypes of Csk-knockdown cells with control, we analyzed the electroporated brains at E16 and E17, when control cells are in the midst of migrating in the IZ or CP. We found small cell clusters in the IZ of the Csk-sh905–electroporated cerebral cortices at both E16 and E17, similar to the P0 cortices (Fig. 4, *E* and *F*). The ratio of single cells without attachment to neighboring cells was decreased in the Csk-sh905–electroporated cerebral cortices, and the ratio of cells in two cell clusters was increased (Fig. 4*G*). Considering that CA-Fyn-expressing cells also exhibited a similar increase of intercellular attachments (Fig. 1*I*), these results suggest that Csk knockdown–mediated local enhancement of the Fyn and Src activities increases the attachment between immature neurons. Moreover, many Csk-knockdown cells with round morphologies were observed in the lower IZ at both E16 and E17, whereas most of the control cells exhibited a multipolar morphology (Fig. 4, *E* and *H*). The ratio of round cells without immature neurites to total GFP-positive cells was increased in the IZ of the Csk-sh905–electroporated cerebral cortices, compared with control, and the ratio of multipolar cells was significantly decreased (Fig. 4*I*). Similar to CA-Fyn-electroporated cells in the IZ, the number and average length of the immature neurites of Csk-knockdown cells in the IZ were reduced (Fig. 4, *J* and *K*). These data indicate that overactivation of the downstream signaling pathways of SFKs by CA-Fyn expression or Csk knockdown increases the attachment between immature neurons and suppresses immature neurite formation.

### Csk is dispensable for neural progenitor proliferation and differentiation

Because Csk exhibits tumor-suppressing properties, we next examined the effects of Csk knockdown–mediated overactivation of SFKs on neural progenitor proliferation. Csk-sh905 was electroporated into E14 mouse embryonic cerebral cortices, and the electroporated brains were fixed at E15, 1 day after the electroporation. EGFP-positive cells were mainly observed in the VZ, where the neural progenitors are located, in both control and Csk-knockdown cerebral cortices at E15 (Fig. 5*B*). To detect proliferating neural progenitors, we performed immunohistochemistry for phospho-Histone H3 (PH3), a marker for M phase in cell cycle. The ratio of PH3-positive proliferating neural progenitors to GFP-positive electroporated cells in the VZ was comparable between control and Csk-knockdown cerebral cortices (Fig. 5, *A*–*C*), suggesting that Csk does not function as a tumor suppressor in neural progenitors.

Neural progenitors in the VZ generate immature neurons, which become positive for Tuj1, a neuronal marker. We stained E16 cerebral cortices for Tuj1 and found that the ratio of Tuj1-positive immature neurons to GFP-positive electroporated cells in the IZ was unchanged between control and the Csk-knockdown cerebral cortices (Fig. 5, *D*–*F*), indicating that Csk knockdown affects neither neural progenitor proliferation nor neuronal differentiation. It further suggests that the migration defects of Csk-knockdown cells are not derived from abnormal proliferation and/or differentiation of neural progenitors, which is consistent with our results showing that a neuron-specific Tα1 promoter–driven Csk can rescue the migration defects of Csk-knockdown cells (Figs. 4, *A*, *B* and S4, *C*, *D*).

### Membrane-bound and cytosolic Fyn may have different functions

**Figure 6:**
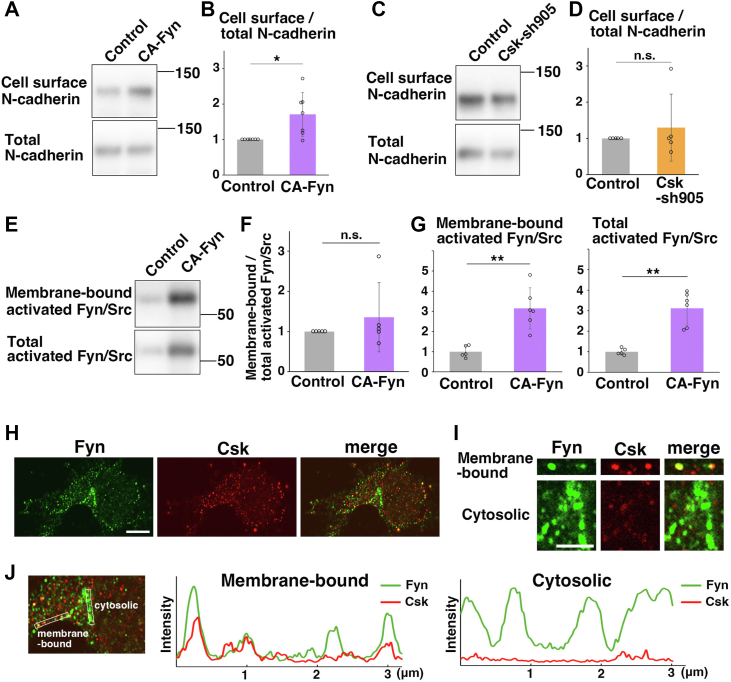
**Fyn, but not Csk, exhibits cytosolic localization and regulates the cell surface levels of N-cadherin.***A*–*G*, primary cortical neurons from E15 cerebral cortices were transfected with the indicated plasmids, incubated for 2 days *in vitro*, and subjected to cell surface biotinylation assays (see the Experimental procedures section). Immunoblot analyses of the precipitates (biotinylated cell surface proteins and their associated membrane-bound proteins) and total cell lysates with anti-N-cadherin (*A*–*D*) or antiactivated Fyn/Src (Y416P-Src) (*E*–*G*) antibodies were performed. The *graphs* in (B) and (*D*) show the mean ratios of immunoblot band intensities of cell surface N-cadherin to that of total N-cadherins ± SD with the individual points. Significance was determined by the one-sample *t* test. CA-Fyn in (*B*): *p* = 0.0227 (n = 7 biological replicates). Csk-sh905 in (*D*): *p* = 0.509 (n = 5 biological replicates) ns, no significant differences, ∗*p* < 0.05. The *graph* in (*F*) shows the mean ratios of immunoblot band intensities of membrane-bound activated Fyn/Src to that of total activated Fyn/Src ± SD with the individual points. Significance was determined by the one-sample *t* test. CA-Fyn in (*F*): *p* = 0.408 (n = 5 biological replicates). ns, no significant differences. The *graphs* in (*G*) show the mean ratios of immunoblot band intensities of activated Fyn/Src in CA-Fyn-expressing cells, compared with control cells, ± SD with the individual points. Each value in the *graphs* in (*G*) was normalized with the means of the control data. Control: n = 5 biological replicates, CA-Fyn: 6 biological replicates. Significance was determined by Welch’s *t* test. Membrane-bound activated SFKs in CA-Fyn-expressing cells in (*G*): *p* = 0.00270, total activated SFKs in CA-Fyn-expressing cells in (*G*): *p* = 0.00115. ∗∗*p* < 0.01. *H*–*I*, primary cortical neurons from E15 cerebral cortices incubated for 2 days *in vitro* and stained with the indicated antibodies. *Upper and lower panels* in (*I*) are high-magnification images of the plasma membrane or perinuclear regions, respectively. The *graphs* in (*J*) show the fluorescence intensities of Fyn or Csk on the lines indicated in the *left panel*. The fluorescence intensities were measured by ImageJ. The scale bars represent 5 μm (*H*) and 2 μm (*I*). CA-Fyn, constitutive active form of Fyn; Csk, C-terminal Src kinase; E15, embryonic day 15; SFK, Src family kinase.

Given that overactivation of Fyn/Src promoted the attachment between immature neurons without affecting neural progenitor proliferation and differentiation, we next analyzed the cell surface levels of cell adhesion molecules. As we previously showed that N-cadherin, a homophilic cell-to-cell adhesion molecule, is essential for cortical neuronal migration (45, 46), we measured the cell surface levels of N-cadherin by using membrane-impermeable sulfo-NHS–biotin. We found that the cell surface levels of N-cadherin were increased in CA-Fyn-transfected neurons, compared with control (Fig. 6, *A* and *B*). These data indicate that overactivation of Fyn promotes cell surface expression of N-cadherin in cortical neurons, in contrast to previous reports that overactivation of Src or Fyn induces internalization of cadherins, including N-cadherin, resulting in downregulation of cell surface N-cadherin (8, 11, 12, 13).

**Supporting Figure S6:**
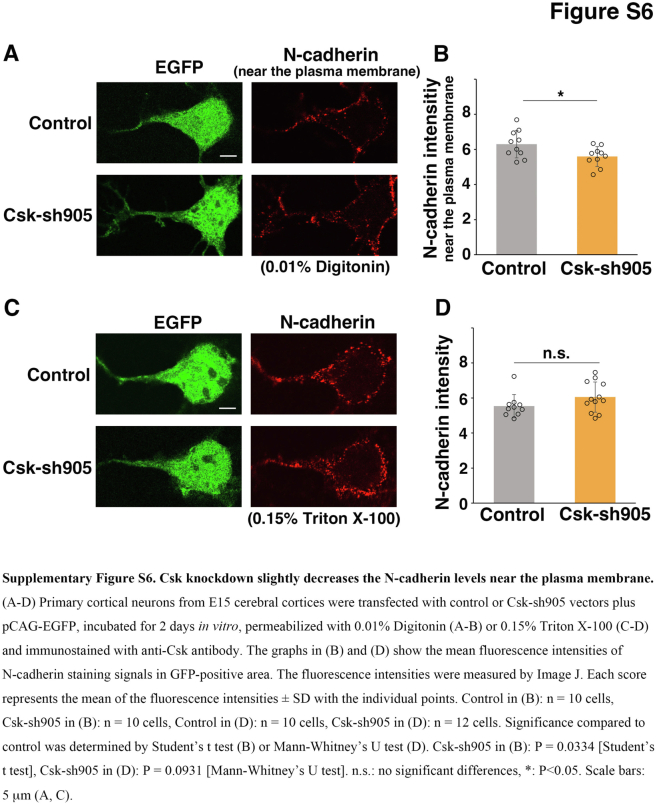


We next performed similar experiments with Csk-knockdown neurons. However, the cell surface levels of N-cadherin were not significantly changed between control and Csk-sh905-expressing neurons (Fig. 6, *C* and *D*). When N-cadherin was immunostained in primary cortical neurons that were permeabilized with 0.15% Triton X-100, the strength of N-cadherin signals was not changed in control and Csk-knockdown neurons, suggesting that total N-cadherin levels are unchanged (Fig. S6, *C* and *D*). In contrast, when primary cortical neurons were permeabilized with 0.01% digitonin, a weaker detergent that allows us to detect only the proteins localized near the plasma membrane (47), N-cadherin was slightly decreased in the Csk-knockdown neurons (Fig. S6, *A* and *B*), suggesting that the N-cadherin in the vesicles or endosomes near the plasma membrane was affected in the Csk-knockdown neurons.

Because CA-Fyn increased N-cadherin cell surface levels while Csk knockdown did not, we compared their Fyn/Src activation patterns. As described above (Fig. 3, *D* and *E*), Csk knockdown only affected SFK activities near the plasma membrane. In contrast, the ratio of membrane-bound–activated Fyn/Src to total activated Fyn/Src was unchanged in the CA-Fyn-expressing neurons (Fig. 6, *E* and *F*), but CA-Fyn expression strongly increased both membrane-bound and total Fyn/Src activities (Fig. 6*G*), suggesting that CA-Fyn heavily phosphorylates Fyn/Src substrates not only near the plasma membrane but also in the cytoplasm. These data indicate that Csk knockdown promotes Fyn/Src activities only near the plasma membrane (membrane-bound Fyn/Src), which is not sufficient to increase the cell surface levels of N-cadherin, whereas increased activities of cytosolic Fyn may be required for cell surface recruitment of N-cadherin, independent of Csk.

We next compared the subcellular localization of endogenous Fyn and Csk in primary cortical neurons and found that they were colocalized near the plasma membrane (Fig. 6*H*, *upper panels* in Fig. 6*I* and *left graph* in Fig. 6*J*), consistent with our results showing that both Csk knockdown and CA-Fyn increased SFK activities near the plasma membrane. Interestingly, we also observed additional perinuclear staining for Fyn but not Csk (*lower panels* in Fig. 6*I* and *right graph* in Fig. 6*J*). The perinuclear Fyn was partially colocalized with GM130, a marker for *cis*-Golgi (Fig. S7, *A* and *B*), suggesting that cytosolic Fyn may have a Csk-independent function at the *cis*-Golgi.

We previously reported that increase of cell-to-cell adhesion by overexpression of N-cadherin disturbs neuronal migration (48). N-cadherin-overexpressing neurons tightly attach to the radial fiber, and the distance between the center of the nuclei of N-cadherin-overexpressing neurons and the radial fiber is decreased (48) (Fig. S7, *C* and *D*). However, unlike N-cadherin overexpression, CA-Fyn and Csk knockdown did not affect the distance between their nuclei and the radial fibers (Fig. S7, *C* and *D*). CA-Fyn-expressing neurons form cell aggregates in the lower-IZ before the radial fiber–dependent migration, and these extra intercellular adhesions in the lower-IZ may perturb the pia-directed migration before attachment with the radial fibers.

### Csk regulates cell surface levels of L1-CAM

**Figure 7:**
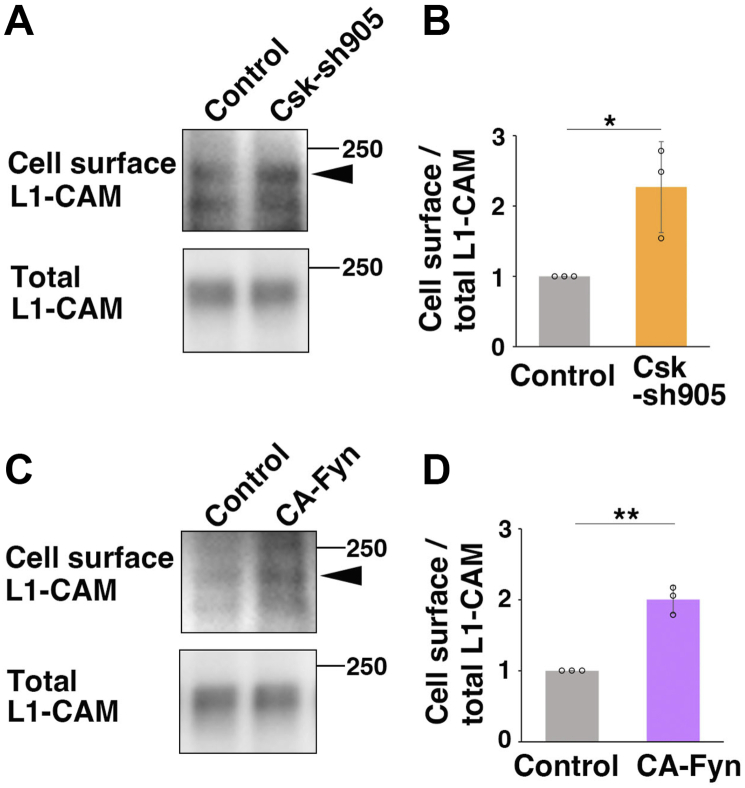
**Both CA-Fyn and Csk knockdown increase the cell surface levels of L1-CAM.***A*–*D*, primary cortical neurons from E15 cerebral cortices were transfected with the indicated plasmids, incubated for 2 days *in vitro*, and subjected to cell surface biotinylation assays (see the Experimental procedures section). Immunoblot analyses of the precipitates (biotinylated cell surface proteins and their associated membrane-bound proteins) and total cell lysates with anti-L1-CAM antibody were performed. The *graphs* in (*B*) and (*D*) show the mean ratios of immunoblot band intensities of cell surface L1-CAM to that of total L1-CAM ± SD with the individual points. Significance was determined by the one-sample *t* test. Csk-sh905 in (*B*): *p* = 0.0261 (n = 3 biological replicates). CA-Fyn in (*D*): *p* = 0.00322 (n = 3 biological replicates). ∗*p* < 0.05, ∗∗*p* < 0.01. CA-Fyn, constitutive active form of Fyn; Csk, C-terminal Src kinase; E15, embryonic day 15.

Given that increased N-cadherin could not explain the small cluster formation of Csk-knockdown immature neurons, we examined other cell adhesion molecules that are upregulated in both CA-Fyn-expressing and Csk-knockdown neurons. We focused on L1-CAM, an immunoglobulin superfamily cell adhesion molecule that is required for cortical neuronal migration (46). Our cell surface biotinylation assays revealed that cell surface levels of L1-CAM were increased in CA-Fyn-expressing neurons and Csk-knockdown neurons (Fig. 7, *A*–*D*). These data suggest that increased L1-CAM strengthens the attachments between immature neurons in the CA-Fyn overexpression and Csk knockdown.

## Discussion

Cell adhesion is essential for development and maintenance of multicellular organisms, but the regulatory mechanisms of cell adhesion *in vivo* are poorly understood. Although overactivation of Src or Fyn is reported to disrupt cell-to-cell adhesion by inducing cadherin internalization in cultured cells (7, 8, 9, 10, 11, 12, 13, 14), our results indicate that constitutive activation of Fyn, an SFK that is abundantly expressed in brains, promotes cell surface expression of N-cadherin and attachment between immature neurons in the IZ of the mouse cerebral cortex. Our previous data indicate that knockdown of Rab21, a small GTPase that mainly regulates caveolin-mediated endocytic pathways, also increases immature neuronal attachment and cell surface levels of N-cadherin (49). However, Rab21-knockdown suppresses immature neurite retraction, whereas CA-Fyn inhibits immature neurite formation, suggesting that Rab21-knockdown and CA-Fyn have opposing effects on immature neuronal morphogenesis. Thus, the mechanisms underlying the increase of cell surface N-cadherin by Rab21-knockdown and CA-Fyn may be different.

How Fyn promotes cell surface levels of N-cadherin remains to be addressed in future studies, but our data show that Fyn is localized at the *cis*-Golgi. Thus, unlike Rab21-knockdown, which disturbs N-cadherin endocytic pathways, Fyn may control N-cadherin trafficking at the Golgi. Because N-myristoylation, an essential lipid modification to localize SFKs at the plasma membrane, is also adapted to Golgi-associated proteins, such as GRASP55 (50, 51), SFKs may localize with Golgi-associated proteins and modulate their activities to regulate the secretory trafficking of transmembrane proteins.

In contrast to CA-Fyn, knockdown of Csk enhances SFK activities only near the plasma membrane, suggesting that Csk knockdown does not affect cytosolic Fyn activities. Our results indicate that Csk knockdown has little effect on the cell surface levels of N-cadherin, which supports our hypothesis that cytosolic Fyn, but not membrane-bound SFKs, may be involved in the enhancement of N-cadherin cell surface levels. A previous study indicated that forced localization of Csk to the plasma membrane reduces SFK activities and cellular invasiveness in SW620 colorectal cancer cells, whereas simple overexpression of Csk increases anchorage-independent growth of SW620 cells in soft agar, similar to overactivation of SFKs, implying that cytosolic Csk may promote oncogenic signals (52). Although we could not observe the perinuclear accumulation of Csk in neurons, cytosolic Csk, as well as Fyn, might have an additional function in cell adhesion and migration.

Despite no significant effect of Csk knockdown on cell surface levels of N-cadherin, our *in vivo* electroporation experiments show that Csk knockdown slightly promotes the attachment between immature neurons. Given that both CA-Fyn and Csk knockdown increase cell surface levels of L1-CAM, this increased L1-CAM-mediated cell adhesion may lead to the small cluster formation in immature neurons, as observed in the Csk knockdown, whereas upregulation of both N-cadherin- and L1-CAM-mediated cell-to-cell adhesion, which occurs in the CA-Fyn overexpression, causes large aggregates of immature neurons.

In addition to the cluster formation, Csk knockdown, as well as CA-Fyn, results in neuronal migration defects. This suggests that Csk-mediated upstream regulation of membrane-bound Fyn/Src provides fine-tuning of immature neuronal adhesion, which may be important for subsequent pia-directed neuronal migration. Consistent with this, previous studies indicate that increased cell-to-cell adhesion disturbs neuronal migration in the developing cerebral cortex (45, 48). In contrast, knockdown of N-cadherin or L1-CAM also results in neuronal migration defects (45, 48, 53). Thus, proper balance in the strength of cell-to-cell adhesion is essential for neuronal migration, and it is regulated in part by SFK activities. Interestingly, Csk knockdown and CA-Fyn also perturb immature neurite formation. Because immature neurite elongation depends on actin reorganization (31, 54), the Csk-Fyn signals may control actin cytoskeletal organization. In fact, it has been reported that Fyn and Src regulate Rho family small GTPases, central regulators for actin reorganization, independent of the regulation of cell adhesion (55, 56, 57).

In many cultured cells, overactivation of Src or Fyn disrupts cadherin-mediated cell-to-cell adhesion. There are differences between previous and current studies, for example, neurons do not form adherens junctions, which are supported by stable circumferential actin bundles (58). Unlike adherens junctions, N-cadherin in migrating neurons is constantly internalized and recycled to the plasma membrane (45). Therefore, regulatory mechanisms of cadherin dynamics might differ between neurons and epithelial cells. Our data suggest that activation of membrane-bound SFKs by Csk knockdown does not change the cell surface levels of N-cadherin but slightly decreases the N-cadherin near the plasma membrane. It is possible that the effect of membrane-bound SFKs on cell adhesion may potentially downregulate N-cadherin, but this effect may be much weaker in neurons, compared with epithelial cells. When CA-Fyn is expressed, the cytosolic Fyn may significantly contribute to increase of the N-cadherin on the plasma membrane, whereas membrane-bound SFKs have little effect on N-cadherin dynamics. Further research is needed to investigate the roles of cytosolic Fyn and the mechanisms regulating the balance between membrane-bound and cytosolic Fyn and Src.

Fyn and Src regulate both cell-to-cell and cell-to-ECM adhesion through the phosphorylation of many substrates, such as cadherin–catenin complex and focal adhesion kinase, respectively (11, 16, 59). Cell-to-ECM adhesion plays important roles in the migration of many cell types (60). However, our previous studies indicate that β1-integrin–mediated cell-to-ECM adhesion is only required for the terminal translocation, but not the early and middle phases of neuronal migration, whereas N-cadherin-mediated cell-to-cell adhesion plays essential roles in the early and middle phases of neuronal migration, including the multipolar-to-bipolar transition and the locomotion mode of long-distance migration (39, 45, 61). Therefore, the phenotypes of Csk-knockdown neurons in the IZ may not result from defects in cell-to-ECM adhesion.

In addition to the activation of SFKs, suppression of SFK activities by treatment with PP2, an inhibitor for SFKs, or *in vivo* knockdown of Fyn, inhibits neuronal migration (27), suggesting that both the increase and decrease of SFK activities disturb the early phase of neuronal migration. On the other hand, SFKs are also reported to have an important function in the final phase of neuronal migration, in cooperation with reelin–Dab1 signaling (62). Under the control of reelin, Dab1 is phosphorylated by Fyn and Src, which is essential for the induction of downstream signaling pathways, including Rap1-dependent activation of α5β1-integrins (36, 37, 38, 39). Whereas Fyn knockout mice exhibit abnormal neuronal positioning, double knockout of Fyn and Src results in roughly inverted cortical layering and defects in preplate splitting, both of which are hallmarks of reelin- or dab1-deficient brains (26). Interestingly, the association of reelin–Dab1 signaling and Golgi dynamics has been reported (63, 64). Reelin increases protein levels of GRASP65 and GRASP55, which are required for neuronal migration and dendrite morphogenesis (65). It is thus possible that Fyn or other SFKs may control Golgi function partly through reelin–Dab1 signaling.

In contrast to postmitotic neuronal migration, neural progenitor proliferation and differentiation into neurons are not visibly affected in the Csk-knockdown cells, although Csk has tumor-suppressing activity in many non-neuronal cells (22, 23, 66). It is consistent with our immunohistochemical analyses showing weak expression of Csk in the VZ, where neural progenitors proliferate. Moreover, Fyn/Src-double knockout mice do not alter the neurogenesis in the VZ (26). In contrast, it has been reported that Fyn and Src are involved in brain tumors, such as glioma (67, 68). Although similarities between glioma and neural development in cell migration have been suggested (69, 70), cell proliferation during normal neurogenesis in developing brains may use different signaling pathways, at least in part, than in glioma in mature brains.

In conclusion, our results indicate that Csk plays pivotal roles in neuronal migration and morphological changes during cerebral cortical development, possibly through fine-tuning of cell-to-cell adhesion. Csk suppresses overactivation of membrane-bound SFKs, which contributes to proper regulation of cell surface levels of L1-CAM. Our data also suggest a Csk-independent function of cytosolic Fyn in cell adhesion. Thus, SFKs have multiple functions at both subcellular and tissue levels, and therefore, their activities should be tightly and locally controlled by Csk and other upstream regulators to exert a multistep developmental program in the mammalian cerebral cortex.

## Experimental procedures

### Antibodies and chemical reagents

Primary antibodies used in this study were anti-Csk (610079; BD Biosciences), anti-Y530P-Src (2105; Cell Signaling Technology), anti-Y416P-Src (6943; Cell Signaling Technology), anti-total-Src (ab231081; abcam [Fig. S2*A*]; 2123, Cell Signaling Technology [Fig. 3*B*]), anti-Fyn (ab125016; Abcam), anti-Pag1 (25029-1-AP; Proteintech), anti-N-cadherin (13116, Cell Signaling Technology; 14215, Cell Signaling Technology [Fig. S6]), anti-L1-CAM (ab24345; abcam), anti-GFP (AB16901; Merck), anti-PH3 (9701; Cell Signaling Technology), anti-βIII tubulin (Tuj1) (801202; BioLegend), anti-Phospho-Tyrosine (pY1000) (8954; Cell Signaling Technology), anti–focal adhesion kinase (ab40794; Abcam), anti-GM130 (610822; BD Biosciences), anti-Cux1 (sc-13024; Santa Cruz), anti-Ctip2 (ab18465; Abcam), Nestin (556309; BD Biosciences), and anti-β-tubulin (T5201; Sigma) antibodies. Regarding antibodies for phosphorylated Src, anti-Y530P-Src and anti-Y416P-Src antibodies recognize the Src phosphorylated at the C-terminal region (Tyr535 in mouse Src [isoform 1]; corresponding to Tyr530 in human and Tyr527 in chicken) and the activated Src with autophosphorylation (Tyr424 in mouse Src [isoform 1]; corresponding to Tyr419 in human and Tyr416 in chicken), respectively. Both anti-Y530P-Src and anti-Y416P-Src antibodies crossreact with Fyn (Fig. S2) and may also crossreact with other SFKs, including Yes (according to the datasheets of Cell Signaling Technology). 4′,6-Diamidino-2-phenylindole dihydrochloride (DAPI) solution was purchased from Wako (340-07971) and Nacalai Tesque (19178-91). EZ-Link Sulfo-NHS–Biotin was purchased from Thermo Fisher Scientific (21217).

### Plasmids

Plasmids were prepared using the EndoFree plasmid purification kit (Qiagen) or NucleoBond Xtra Midi (Takara). Human Csk complementary DNA was purchased from DNAFORM (ID: 6526810) and inserted into pTα1-MCS1 (45), by using In-Fusion HD cloning kit (Clontech), to generate Tα1-wt-Csk. CA-Fyn (Y531F-human c-Fyn) and wt-Fyn complementary DNAs were kindly provided by Dr Shigeru Kanda (71) and inserted into pCAG-MCS2 (72) to generate CAG-CA-Fyn and CAG-wt-Fyn, respectively. pCAG-EGFP and CAG-N-cadherin were described previously (40, 45).

To construct shRNA-expressing vectors, oligonucleotides targeting the *Csk* coding sequence (Csk-sh905: 5′-TGGAGTACCTGGAGGGTAA-3′, Csk-sh1310: 5′-GGGAACAGCTCGAGCACAT-3′) and their complementary sequences were inserted into the pSilencer 3.1-H1 vector (Ambion). All contain a hairpin loop sequence (5′-TTCAAGAGA-3′). These sequences were designed based on information from shRNA sequence analyses (B-Bridge International, Inc). A control vector containing a scrambled nontargeting sequence was purchased from Ambion.

### Animal experiments

All animal experiments were approved by and performed in accordance with the guidelines established by Kyoto University, Tohoku Medical and Pharmaceutical University, and Keio University. Pregnant ICR mice were purchased from SLC Japan. All mice were housed and maintained under specific pathogen–free conditions.

### *In utero* electroporation

*In utero* electroporation experiments were performed as described previously with minor modifications (40, 73). All electroporations in this report were performed on E14 embryos. Pregnant mice were deeply anesthetized, and an abdominal incision was made to access the uterus. Approximately 1 μl of plasmid DNA (Csk-sh905, Csk-sh1310, CA-Fyn, and control vectors: 1 μg/μl, N-cadherin: 10 μg/μl, Tα1-wt-Csk: 0.02 μg/μl, and pCAG-EGFP: 1 μg/μl) in endotoxin-free TE buffer (Qiagen) containing Fast Green was injected into the lateral ventricle of embryonic brains with a glass micropipette (G-1 or GD-1; Narishige). Holding the embryo *in utero* with forceps-type electrodes (NEPA GENE or BEX), 50 ms electric pulses of 35 V were delivered five times at intervals of 450 ms with a square electroporator (NEPA21 [NEPA GENE] or CUY21EDIT [NEPA GENE] or CUY21 [BEX]). After electroporation, the uterus was placed back into the abdominal cavity, allowing embryos to continue developing. At indicated stages, embryos were harvested, and coronal sections of electroporated brains were prepared by using a cryostat (Leica).

### Immunohistochemistry

Immunohistochemical analyses were performed as described previously with minor modifications (45, 46). Embryonic brains were fixed in 4% paraformaldehyde in PBS for several hours at 4 °C. Frozen cortical sections were washed with PBS, treated with GS-PBS (10% goat serum in PBS) containing 0.05% Triton X-100 for 1 h at room temperature, and subsequently incubated with diluted primary antibodies in GS-PBS containing 0.1% Tween-20 at 4 °C overnight. After three washes in PBS, sections were treated with Alexa488-, Alexa555-, or Alexa647-conjugated secondary antibodies (Molecular Probes) diluted in PBS for 45 min at room temperature, followed by three washes in PBS. The nuclei were stained with DAPI. Fluorescence images were obtained by FV-10i laser scanning confocal microscopy (Olympus) or A1R laser scanning confocal microscopy with a high-sensitivity GaAsP detector (Nikon) or SpinSR10 spinning disk confocal microscopy (Olympus). For staining with anti-PH3 or anti-βIII-tubulin (Tuj1) antibodies, frozen cortical sections were treated with HistoVT-One (Nacalai Tesque) for 20 min at 70 °C after the fixation.

### Primary cultures, transfection, and immunocytochemistry

Primary cultures of embryonic cortical neurons were performed as described previously with minor modifications (74). E15 mouse embryonic cerebral cortices were treated with 0.25% trypsin–EDTA for 10 to 15 min at 37 °C and dissociated into single cells by gentle trituration. Cells were suspended in 500 to 1200 μl of Neurobasal plus medium (Invitrogen) supplemented with B-27 plus (Invitrogen), 2 mM GlutaMAX (Invitrogen), and 100 units/ml penicillin and 0.1 mg/ml streptomycin (Sigma), and then plated on coverslips, 6 cm dishes, 6-well plates, or 12-well plates coated with 0.1 mg/ml poly-d-lysine (Sigma or MP Biochemicals). Cells were incubated at 37 °C for 2 or 5 days. Transfections into primary cultures of E15 cerebral cortices were performed using the Amaxa mouse neuron nucleofector kit (Lonza) according to the manufacturer’s instructions with some modifications.

For immunocytochemistry (47), cells were fixed with 4% paraformaldehyde in PBS for 20 min, permeabilized with GS-PBS containing 0.15% Triton X-100 for 5 min (or 0.01% digitonin for 1 min in Fig. S6, *A* and *B*), and blocked with GS-PBS for 1 h at room temperature. Primary and secondary antibodies were applied as described above for immunohistochemistry. The nuclei were stained with DAPI. Fluorescence images were obtained by A1R laser scanning confocal microscopy with a high-sensitivity GaAsP detector (Nikon).

### Cell surface biotinylation assay

Primary cultured cortical neurons (2 DIV) were incubated with 0.5 mg/ml Ez-Link Sulfo-NHS–Biotin (Thermo Fisher Scientific) in PBS for 30 min at 4 °C under gentle agitation. After the incubation, cells were washed twice with 50 mM glycine in PBS for quenching the biotinylation reaction. Subsequently, cells were treated with lysis buffer (20 mM Tris–HCl [pH 7.5], 150 mM NaCl, 1% Triton X-100, and EDTA-free Complete protease inhibitor cocktail [Roche], and phosphatase inhibitor cocktail [EDTA free] [Nacalai Tesque]) and harvested with a cell scraper. After 1 h incubation on ice, the lysates were sonicated and centrifuged at 5000*g* for 5 min at 4 °C to remove cell debris.

The clarified supernatant (total lysate) was incubated with a 1% streptavidin–sepharose slurry (GE Healthcare) for 1 h at 4 °C (or 2% streptavidin-magnetic beads [Cytiva] overnight at 4 °C [Fig. 7]). The incubated streptavidin beads were precipitated and thoroughly washed in wash buffer (50 mM Tris–HCl, 150 mM NaCl, and 2 M urea) to purify the solubilized biotinylated proteins. The precipitates (biotinylated cell surface proteins and their binding proteins, which are referred to as “membrane-bound proteins” in this article) were resuspended in SDS sample buffer (50 mM Tris–HCl [pH 6.8], 2% SDS, 10% glycerol, 100 mM DTT, and bromophenol blue) and boiled for 5 min.

The membrane-bound proteins were subjected to immunoblot analyses to detect “membrane-bound activated Fyn and Src” or “cell surface N-cadherin” or “cell surface L1-CAM.” The total lysates before the precipitation with streptavidin beads were also subjected to immunoblot analyses, concomitantly with the membrane-bound proteins (blotting on the same polyvinylidene difluoride membrane), to detect total-activated Fyn/Src or N-cadherin or L1-CAM.

### Immunoblotting

Immunoblot analyses were performed as described previously with minor modifications (49). For preparing cell lysates, primary cultured neurons were washed with ice-cold PBS, treated with lysis buffer (20 mM Tris-HCl [pH 7.5], 150 mM NaCl, 1% Triton X-100, EDTA-free Complete protease inhibitor cocktail [Roche], and phosphatase inhibitor cocktail [EDTA-free] [Nacalai Tesque]) and harvested with a cell scraper. After 1 h incubation on ice, the lysates were sonicated and centrifuged at 5000*g* for 5 min at 4 °C to remove cell debris. The supernatants were mixed with SDS sample buffer (50 mM Tris–HCl [pH 6.8], 2% SDS, 10% glycerol, 100 mM DTT, and bromophenol blue).

Cell lysates in the SDS sample buffer were separated with SDS-PAGE and transferred by electrophoresis onto polyvinylidene difluoride membranes. Membranes were blocked with Blocking One (Nacalai Tesque) for 1 h and probed with primary antibodies in Can Get Signal reagents (TOYOBO). After washing with Tris-buffered saline with Tween-20 (20 mM Tris–HCl [pH 7.5], 150 mM NaCl, and 0.05% Tween-20), the membranes were treated with horseradish peroxidase–conjugated secondary antibodies and ECL Prime Western blotting detection reagents (Amersham) or Chemi-lumi one super (Nacalai Tesque). Signals were detected with a cooled CCD camera (FUSION-FX7.EDGE; Vilber-Lourmat) and measured with ImageJ.

### Immunoprecipitation

Primary cultured cortical neurons (2 DIV) were treated with lysis buffer (1% Triton X-100, 20 mM Tris–HCl [pH 7.5], 150 mM NaCl, EDTA-free Complete protease inhibitor cocktail [Roche], and EDTA-free phosphatase inhibitor cocktail [Nakalai Tesque]) and harvested with a cell scraper. After 1 h incubation on ice, the lysates were sonicated and centrifuged at 5000*g* for 5 min at 4 °C to remove cell debris. The clarified supernatants were incubated with anti-Fyn or anti-Src antibody at 4 °C overnight, followed by additional incubation with protein G magnetic beads (Cytiva) at 4 °C for 60 min. The beads were washed three times with PBS, and bound proteins were separated by SDS-PAGE and subjected to immunoblot analysis as described above.

### Quantitative analysis for the ratio of cells with different morphology

The morphologies of the immature neurons in the IZ were analyzed on frozen sections of the cerebral cortices at E17, 3 days after electroporation. Cells exhibiting bipolar (locomoting neurons) or round or multipolar morphologies were quantified in the IZ. Bipolar (locomoting) cells were defined as cells with a thick and pia-directed leading process. Using our definition, round and multipolar cells do not possess a leading process. Multipolar cells were defined as cells with more than three neurites or polygonal morphology.

### Quantitative analysis for the neuronal positioning

The extent of migration was estimated by counting the number of EGFP-positive cells in distinct regions of the cerebral cortices, as described previously (49). Fluorescence images of the frozen sections of the electroporated brains were captured by SpinSR10 spinning disk confocal microscopy (Olympus). EGFP-positive cells within the same width regions in the layers II–IV, V–VI, IZ, and SVZ/VZ of the cerebral cortices were counted, and the ratios of the cell numbers in each layer to total cell numbers were calculated and plotted in the graphs.

### Statistical analyses

Statistical significance was calculated using two-tailed Student’s *t* test (for data showing normal distribution and equality of variance, evaluated by Shapiro–Wilk test and *F* test), Welch’s *t* test (for data showing normal distribution, but not equality of variance, evaluated by Shapiro–Wilk test and *F* test), Mann–Whitney *U* test (for data that are not normal distribution, evaluated by Shapiro–Wilk test), one-sample *t* test (for comparisons to control data normalized to one) or multiple comparison analyses (one-way ANOVA with post hoc Tukey–Kramer test), by using R-package statistical software. A *p* value of <0.05 was considered statistically significant. Sample size and statistical test used for each statistical hypothesis testing are shown in the corresponding figure legends.

## Data availability

All data are contained within the article and the supporting information.

## Supporting information

This article contains supporting information (Figs. S1–S7).

## Conflict of interest

The authors declare that they have no conflicts of interest with the contents of this article.

## Figures and Tables

**Supporting Figure S7:**